# Supplementary material for: Voices of surgical wards nurses on barriers hindering acute post-operative pain management at Tshwane municipality, South Africa
Source: PLoS One. 2025 Feb 21;20(2):e0316809. doi: 10.1371/journal.pone.0316809 (PMC11844844; doi:10.1371/journal.pone.0316809)
Supplement: Attachment — Submitted filename: Plos ONE Review report.docx [file pone.0316809.s002.docx]

Recommendation

Minor Revision

Thank you for sharing your manuscript titled: Voices of surgical wards nurses on barriers hindering acute post-operative pain management at Tshwane municipality, South Africa. An interesting study, a good justification is needed in the ethical consideration section about informed consent.

Below are further feedback for consideration:

**Objective**

I will suggest that you rephrase the objective and use such term as “uncovering”….. instead of “Identifying”

Uncovering barriers that hinder the surgical wards nurses to manage acute postoperative pain at the selected public hospital in Tshwane municipality, Gauteng Province, South Africa.

**Population and sampling**

What are the reasons why other categories are excluded from the study? Are other categories not eligible and why.

You mention professional nurses who are temporary or on contract are not included? Justify why they are excluded?

Could you please rephrase the sentence? (see Manuscript)

**Ethical considerations**

Was the informed consent verbal or written?

If you use AI such as ChatGPT, Please acknowledge it.
