# Supplementary material for: Voices of surgical wards nurses on barriers hindering acute post-operative pain management at Tshwane municipality, South Africa
Source: PLoS One. 2025 Feb 21;20(2):e0316809. doi: 10.1371/journal.pone.0316809 (PMC11844844; doi:10.1371/journal.pone.0316809)
Supplement: Attachment — Submitted filename: MAKOU NM Response to Reviewers.docx [file pone.0316809.s003.docx]

**Rebuttal Letter**

To,

The Editor, Reviewers,

PLOS ONE

Date: 29 August 2024

**Subject: Addressing Editor/Reviewer Comments for PONE-D-24-21430**

Dear Editor, Reviewers,

Thank you for giving us the opportunity to submit a revised manuscript “Voices of surgical wards nurses on barriers hindering acute post-operative pain management at Tshwane municipality, South Africa”. We appreciate the time and effort that you and the reviewers dedicated to providing feedback on our manuscript and are grateful for the insightful comments on and valuable improvements to our paper. We have incorporated most of the suggestions made by the reviewers. Those changes are highlighted within the revised manuscript file with tracked changes. Kindly see below, in blue, for a point-by-point response to the reviewers’ comments and concerns. All page numbers refer to the revised manuscript file with tracked changes.

Thank you. Kind regards,

Authors of **PONE-D-24-21430**

PONE-D-24-21430

Voices of surgical wards nurses on barriers hindering acute post-operative pain management at Tshwane municipality, South Africa

PLOS ONE

Dear Dr. Melia,

Thank you for submitting your manuscript to PLOS ONE. After careful consideration, we feel that it has merit but does not fully meet PLOS ONE’s publication criteria as it currently stands. Therefore, we invite you to submit a revised version of the manuscript that addresses the points raised during the review process.

Please submit your revised manuscript by Sep 27 2024 11:59PM. If you will need more time than this to complete your revisions, please reply to this message or contact the journal office at [plosone@plos.org](mailto:plosone@plos.org). When you're ready to submit your revision, log on to <https://www.editorialmanager.com/pone/> and select the 'Submission Needing Revision' folder to locate your manuscript file.

Please include the following items when submitting your revised manuscript:

- A rebuttal letter that responds to each point raised by the academic editor and reviewer(s). You should upload this letter as a separate file labeled 'Response to Reviewers'.

Author response: Included.

- A marked-up copy of your manuscript that highlights changes made to the original version. You should upload this as a separate file labeled 'Revised Manuscript with Track Changes'.

Author response: Included.

- An unmarked version of your revised paper without tracked changes. You should upload this as a separate file labeled 'Manuscript'.

Author response: Included.

If you would like to make changes to your financial disclosure, please include your updated statement in your cover letter. Guidelines for resubmitting your figure files are available below the reviewer comments at the end of this letter.

If applicable, we recommend that you deposit your laboratory protocols in [protocols.io](http://protocols.io/) to enhance the reproducibility of your results. Protocols.io assigns your protocol its own identifier (DOI) so that it can be cited independently in the future.

For Instructions see: [https://journals.plos.org/plosone/s/submission-guidelines#loc-laboratory-protocols](https://c05y1x9s.r.us-east-2.awstrack.me/L0/https:%2F%2Fjournals.plos.org%2Fplosone%2Fs%2Fsubmission-guidelines%23loc-laboratory-protocols/1/010f01914c9b0e50-316e2212-1114-4f02-8b57-ba68278cac14-000000/k2HjCDvrV3m-oRP0v5C3PPKctcc=172). Additionally, PLOS ONE offers an option for publishing peer-reviewed Lab Protocol articles, which describe protocols hosted on [protocols.io](http://protocols.io/).

Read more information on sharing protocols at <https://plos.org/protocols?utm> medium=editorial-email&utm source=authorletters&utm campaign=protocols.

We look forward to receiving your revised manuscript.

Kind regards,

Sunil Shrestha
Academic Editor
PLOS ONE

Journal Requirements:

When submitting your revision, we need you to address these additional requirements.

1. Please ensure that your manuscript meets PLOS ONE's style requirements, including those for file naming. The PLOS ONE style templates can be found at
   <https://journals.plos.org/plosone/s/file?id=wjVg/PLOSOne_formatting_sample_main_body.pdf> and 
   <https://journals.plos.org/plosone/s/file?id=ba62/PLOSOne_formatting_sample_title_authors_affiliations.pdf>

Author response: We have followed the above-mentioned PLOS ONE style templates.

2. Please update your submission to use the PLOS LaTeX template. The template	and more information on our requirements for LaTeX submissions can be found	at <http://journals.plos.org/plosone/s/latex>."

Author response: We have followed the PLOS One template available at	<https://journals.plos.org/plosone/s/latex>.

3. Please amend the manuscript submission data (via Edit Submission) to include	authors Dr. Rasweswe Melitah Molatelo and Dr. Mooa Ramadimetja Shirley.

Author response: We have included the above-mentioned authors.

Additional Editor Comments:
Major Revision

[Note: HTML markup is below. Please do not edit.]

Reviewers' comments:

Reviewer's Responses to Questions

**Comments to the Author**

1. Is the manuscript technically sound, and do the data support the conclusions?

The manuscript must describe a technically sound piece of scientific research with data that supports the conclusions. Experiments must have been conducted rigorously, with appropriate controls, replication, and sample sizes. The conclusions must be drawn appropriately based on the data presented.

Reviewer #1: Yes

Author response: We appreciate the reviewer’s feedback, Thank you!

2. Has the statistical analysis been performed appropriately and rigorously?

Reviewer #1: N/A

Author response: Thank you for pointing this out. The reviewer is correct, as our manuscript is based on the qualitative research approach.

3. Have the authors made all data underlying the findings in their manuscript fully	available?

The [PLOS Data policy](http://c05y1x9s.r.us-east-2.awstrack.me/L0/http:%2F%2Fwww.plosone.org%2Fstatic%2Fpolicies.action%23sharing/1/010f01914c9b0e50-316e2212-1114-4f02-8b57-ba68278cac14-000000/0uqKlgmocrdLqalHNcM_0NDn1Co=172) requires authors to make all data underlying the findings described in their manuscript fully available without restriction, with rare exception (please refer to the Data Availability Statement in the manuscript PDF file). The data should be provided as part of the manuscript or its supporting information, or deposited to a public repository. For example, in addition to summary statistics, the data points behind means, medians and variance measures should be available. If there are restrictions on publicly sharing data—e.g. participant privacy or use of data from a third party—those must be specified.

Reviewer #1: Yes

Author response: We appreciate the reviewer’s feedback, Thank you!

4. Is the manuscript presented in an intelligible fashion and written in standard	English?

PLOS ONE does not copyedit accepted manuscripts, so the language in submitted articles must be clear, correct, and unambiguous. Any typographical or grammatical errors should be corrected at revision, so please note any specific errors here.

Reviewer #1: Yes

Author response: We appreciate the reviewer’s feedback, Thank you!

5. Review Comments to the Author

Please use the space provided to explain your answers to the questions above. You may also include additional comments for the author, including concerns about dual publication, research ethics, or publication ethics. (Please upload your review as an attachment if it exceeds 20,000 characters)

**Reviewer #1: Recommendation**
**Minor Revision**

Thank you for sharing your manuscript titled: Voices of surgical wards nurses on barriers hindering acute post-operative pain management at Tshwane municipality, South Africa. An interesting study, a good justification is needed in the ethical consideration section about informed consent.

Author response: We appreciate the reviewer positive encouraging comment.

**Below are further feedback for consideration:**

**Objective**:
I will suggest that you rephrase the objective and use such term as “uncovering”….. instead of “Identifying”

Uncovering barriers that hinder the surgical wards nurses to manage acute postoperative pain at the selected public hospital in Tshwane municipality, Gauteng Province, South Africa.

Author response: As suggested by the reviewer, we have rephrased the objective and the term “uncovering” was used and the changes are highlighted with colour ‘blue’ in line 33, page 2 of the 'Revised Manuscript with Track Changes'.

**Population and sampling:**
What are the reasons why other categories are excluded from the study? Are other categories not eligible and why.

Author response: We appreciate and respect the reviewer’s feedback. Other nursing categories we excluded because the registered professional nurses, registered by a nursing regulatory authority South African Nursing Council are have more knowledge, able to provide supervision and, be responsible and accountable for the delegated nursing activities to the subordinates during the rendering of quality nursing care to the post-operative patients. All other categories are eligible to render quality nursing care to the post-operative patients as they have undergone education and training in the accredited universities and nursing education institutions. However, in this study we were focusing on the registered professional nurses who are able to provide supervision, able to implement, monitor and evaluate post-operative patients and as well as to develop, implement, monitor and evaluate the in-service education program regarding care of the post-operative patients to increase satisfaction and reduce long of hospital stays. Sentence not changed, highlighted with colour ‘blue’ in line 149, page 7 of the 'Revised Manuscript with Track Changes'.

You mention professional nurses who are temporary or on contract are not included? Justify why they are excluded?

Author response: Thank you for pointing this out. We believed that the professional nurses who are on temporary or contract work do not have a permanent job in the public hospital's surgical wards. In this study, we were focusing on the professional nurses, registered by a nursing regulatory authority South African Nursing Council working in the surgical wards of the selected public hospital and have more than twelve months working experience. Sentence rephrased and highlighted with colour ‘blue’ in line 150, page 7 of the 'Revised Manuscript with Track Changes'.

Could you please rephrase the sentence? (see Manuscript)

Author response: We appreciate the reviewer’s feedback. We have added the rephrased sentence and highlighted with colour ‘blue’ in line 150, page 7 of the 'Revised Manuscript with Track Changes' as suggested by the reviewer.

Sentence was: Professional nurses who were on temporary or contract work were also excluded.

Rephrased sentence: In addition, participants in this study were not registered professional nurses employed on a contract or temporary basis in the public hospital's surgical wards.

**Ethical considerations:**
Was the informed consent verbal or written?

Author response: We appreciate the reviewer’s feedback. A written informed consent was obtained prior to participation. Once the participant agreed to take part in the study were given a written informed consent, signed it to avoid coercion. Sentence not changed, highlighted with colour ‘blue’ in line 162, page 7 of the 'Revised Manuscript with Track Changes'.

If you use AI such as ChatGPT, Please acknowledge it.

Author response: We would like to appreciate QuillBot AI, for assisting with rephrasing of sentences.

6. PLOS authors have the option to publish the peer review history of their article ([what does this mean?](https://c05y1x9s.r.us-east-2.awstrack.me/L0/https:%2F%2Fjournals.plos.org%2Fplosone%2Fs%2Feditorial-and-peer-review-process%23loc-peer-review-history/1/010f01914c9b0e50-316e2212-1114-4f02-8b57-ba68278cac14-000000/4mOI8YUQfa9zlwOspLKfy_Rh204=172)). If published, this will include your full peer review and any attached files.

If you choose “no”, your identity will remain anonymous but your review may still be made public.

**Do you want your identity to be public for this peer review?** For information about this choice, including consent withdrawal, please see our [Privacy Policy](https://c05y1x9s.r.us-east-2.awstrack.me/L0/https:%2F%2Fwww.plos.org%2Fprivacy-policy/1/010f01914c9b0e50-316e2212-1114-4f02-8b57-ba68278cac14-000000/AXVQPZ-cJ6F26ZLbnl6VqOX8ImY=172).

Reviewer #1: No

[NOTE: If reviewer comments were submitted as an attachment file, they will be attached to this email and accessible via the submission site. Please log into your account, locate the manuscript record, and check for the action link "View Attachments". If this link does not appear, there are no attachment files.]

While revising your submission, please upload your figure files to the Preflight Analysis and Conversion Engine (PACE) digital diagnostic tool, [https://pacev2.apexcovantage.com/](https://c05y1x9s.r.us-east-2.awstrack.me/L0/https:%2F%2Fpacev2.apexcovantage.com%2F/1/010f01914c9b0e50-316e2212-1114-4f02-8b57-ba68278cac14-000000/oGiYS6UAvCvy2VvvjswEcEUl_r0=172). PACE helps ensure that figures meet PLOS requirements. To use PACE, you must first register as a user. Registration is free. Then, login and navigate to the UPLOAD tab, where you will find detailed instructions on how to use the tool. If you encounter any issues or have any questions when using PACE, please email PLOS at [figures@plos.org](mailto:figures@plos.org). Please note that Supporting Information files do not need this step.

Sincerely yours

The authors
